# ANGPTL4 Expression in Ovarian Granulosa Cells Is Associated With Polycystic Ovary Syndrome

**DOI:** 10.3389/fendo.2021.799833

**Published:** 2022-01-24

**Authors:** Qi Jiang, Ye Pan, Ping Li, Yanjun Zheng, Yuehong Bian, Wenqi Wang, Guihua Wu, Tian Song, Yuhua Shi

**Affiliations:** ^1^ Center for Reproductive Medicine, Cheeloo College of Medicine, Shandong University, Jinan, China; ^2^ Key Laboratory of Reproductive Endocrinology of Ministry of Education, Shandong University, Jinan, China; ^3^ Shandong Key Laboratory of Reproductive Medicine, Jinan, China; ^4^ Shandong Provincial Clinical Research Center for Reproductive Health, Jinan, China; ^5^ National Research Center for Assisted Reproductive Technology and Reproductive Genetics, Jinan, China; ^6^ Women and Children's Hospital, School of Medicine, Xiamen University, Xiamen, China; ^7^ Shandong Provincial Hospital Affiliated to Shandong First Medical University, Jinan, China; ^8^ Guangdong Provincial People's Hospital, Guangdong Academy of Medical Sciences, Guangdong, China

**Keywords:** polycystic ovary syndrome, angiopoietin-like protein 4, mRNA, ovarian granulosa cell, glycolipid metabolism

## Abstract

**Objectives:**

This study aims to characterize the expression of ANGPTL4 in ovarian granulosa cells (GCs) and its association with polycystic ovary syndrome (PCOS).

**Methods:**

This study included 104 PCOS patients and 112 women in control group undergoing *in vitro* fertilization-embryo transfer (IVF-ET) from the reproductive hospital affiliated with Shandong University from 2019 to 2021. By reverse transcription and real-time quantitative (RT-q) PCR, the mRNA expression of ANGPTL4 in GCs was assessed, and clinical information for these patients were then reviewed and analyzed.

**Results:**

The RT-qPCR results showed that ANGPTL4 expression in the control group was significantly lower than that in the PCOS group (*p* = 0.000) and had positive association with AMH (*r* = 0.211), HOMA-IR (*r* = 0.174), LDL/HDL (*r* = 0.176), ApoB/ApoAI (*r* = 0.155), and TC/HDL (*r* = 0.189). Additionally, the high expression of ANGPTL4 in the ovarian granulosa cells might be an independent predictor in PCOS (OR: 3.345; 95% CI: 1.951–5.734) with a close contact with incidence of PCOS (AUC: 0.704; 95% CI: 0.633–0.774, *p* < 0.001).

**Conclusions:**

Our study revealed higher ANGPTL4 expression in ovarian GCs with PCOS. Its association with glucose and lipid metabolism showed that ANGPTL4 might play an important role in PCOS metabolism and pathogenesis.

## Introduction

Polycystic ovary syndrome (PCOS) is one of the most common and complicated endocrine and metabolic disorders, affecting about 6%–20% women of reproductive age (1). In addition to reproductive dysfunction, PCOS can also manifest abnormal glycolipid metabolism while the insulin resistance (IR) is an independent risk factor for several metabolic abnormalities, including dyslipidemia, impaired glucose tolerance, cardiovascular disease, and metabolic syndrome (MetS) (2–4). Throughout the process of oocyte development, there is an interdependence between oocytes and its surrounding granulosa cells providing growth regulators and nutrients. The oocyte in turn promotes growth and differentiation of the granulosa cells. For patients with PCOS, the associated endocrine and glycolipid metabolic disorder can disrupt the interaction between them and then leads to the block of follicular growing, a decrease of high-quality embryos and even lower transplantation rate (5, 6). In recent years, research on relationship between glycolipid metabolism and ovarian function has received increasing awareness. A series of evidence have been provided that lipid metabolism in GCs is essential for maintaining ovarian follicle development in humans (7, 8). However, the mechanisms underlying these associations have not been fully investigated, especially the origins of the metabolic alterations for PCOS as well as new biomarkers.

ANGPTL4 is a member of the angiopoietin-like protein family and is known as a regulator of lipid and glucose metabolism. It is proven to inhibit the activity of lipoprotein lipase (LPL), which hydrolyzes triglyceride (TG) core of TG-rich lipoproteins, chylomicrons, and very low-density lipoproteins (VLDL), and regulates their distribution to peripheral tissues (9–11). Earlier research provided evidence supporting the role of ANGPTL4 in maintaining glucose associated with hyperlipidemia (12). Güneş et al. reported that there was a significant increase of ANGPTL4 level in serum of patients with PCOS compared with healthy population, which showed that ANGPTL4 level in serum may positively correlate with PCOS (13).

Research on ANGPTL4 expression in ovarian granulosa cells (GCs) is still deleted. Therefore, our study aims to investigate the relative expression levels of ANGPTL4 in ovarian granulosa cells in PCOS and examine their possible associations with the glucose and lipid metabolism.

## Materials and Methods

### Patients

In the present study, a total of 104 PCOS patients (PCOS group) and 112 control women (control group) younger than 40 years old who were undergoing their *in vitro* fertilization (IVF) or intracytoplasmic sperm injection (ICSI) cycles were retrospectively recruited in a reproductive hospital affiliated with Shandong University from 2019 to 2021. To diagnose the polycystic ovary syndrome, we referred to the modified Rotterdam criteria (14–16), including oligo- or anovulation combined with either hyperandrogenism or polycystic ovaries. Other causes of hyperandrogenism and ovulation dysfunction were excluded. Inclusion criteria for patients in the control group were as follows: normal menstrual cycle, no endocrine abnormalities, and normal ovarian and uterine morphology confirmed by either ultrasound or histological examination. Additionally, patients were also excluded as follows: recurrent spontaneous abortion, chromosome abnormality for couples, diabetes, adenomyosis, endometriosis, as well as history of ovary surgery. The study is approved by the Ethics Committee of Reproductive Medicine of Shandong University with approval number 58, and signed informed consent has been obtained from all participants.

### Medication Protocol

All the samples received a standardized long-term protocol in mid-luteal phase or gonadotropin-releasing hormone (GnRH) antagonist regimen for ovarian stimulation. For long-term protocol, GnRH agonist (GnRHa) was used to downregulate the function of the pituitary gland on day 21 in the previous menstrual cycle. A dose of recombinant FSH ranging from 75 to 225 IU was initiated after downregulation. As for the GnRH antagonist regimen, recombinant FSH at a dose of 75 to 225 IU was administered on day 3 in the menstrual cycle. GnRH antagonist at a dose of 0.25 mg daily was initiated when at least one follicle reached 12 mm in diameter. For all two protocols, urinary human chorionic gonadotropin (hCG) was intramuscularly injected when at least 2 dominant follicles reached 18–20 mm in diameter. Oocyte retrieval was performed 36–48 h later, and we collected 50 ml follicular fluid meanwhile for ovarian GC isolation.

### Isolation of Ovarian Granulosa Cells

As previously described (17), ovarian GCs were collected from follicular fluid on the oocyte retrieval day, the supernatant of follicular fluid was discarded after centrifuging at 2,000 rcf for 10 min at 4°C, 1 mg of hyaluronidase (Solarbio, Beijing, China) was added per 1 ml of Hanks balanced salt solution (Solarbio, China), and 1 ml of the solution was added to the sample tube. We then incubated the solution at 37°C for 30 min and added it to the upper liquid of 4 ml of human peripheral blood lymphocyte separation solution (TBDscience, Tianjin, China) and avoided mixing with the underlying liquid. We centrifuged the solution at 1,600 rcf for 10 min, collected the interphase, and went through preseparation filters (70 µm) (MACS, Frankfurt am Main, Germany) to eliminate cell clumps that may be inserted into the column. The mixture was centrifuged at 6,000 rcf for 5 min and the supernatant was discarded. We then transferred cells to a new microfuge tube by PBS resuspension and were then centrifuged at 6,000 rcf for 3 min. Fresh GCs were stored at −80°C after discarding the supernatant.

### RNA Extraction, Reverse Transcription, and Quantitative Real-Time PCR

Isolated ovarian GCs were lysed by Trizol reagent (Thermo Fisher Scientific, Inc., Waltham, MA, USA) for 30 min to extract total RNA according to the guidance. The concentration and purity of the total RNA was determined by UV spectrophotometry with the ratio of the optical density being 260/280 nm between 1.8 and 2.1. Total RNA was reverse transcribed into cDNA using Prime Script RT Reagent Kit (TaKaRa, Kusatsu, Japan) for real-time quantitative PCR (RT-qPCR) carried out by TB Green™ Premix Ex Taq™ II (TaKaRa, Japan) and performed on Roche Light Cycle 480 (Hoffman-La Roche, Basel, Switzerland). ANGPTL4 mRNA expression levels were normalized against the corresponding levels of GAPDH mRNA, which served as an internal control and the 2^−ΔΔCt^ method (18) was applied to determine ANGPTL4 expression level. Primer sequences were as follows: ANGPTL4 (forward primer, 5′-TCCTGGACCACAAGCACCTAGAC-3′; reverse primer, 3′-CGGTTGAAGTCCACTGAGCCATC-5′), GAPDH (forward primer, 5′-GCACCGTCAAGGCTGAGAAC-3′; reverse primer, 3′-TGGTGAAGACGCCAGTGGA-5′).

### Statistical Analysis

Data analysis was performed using SPSS (version 20; SPSS Inc., Chicago, IL, USA). Continuous variables of clinical characteristics were displayed as mean ± standard deviation and compared by *t*-test. Parameters abnormally distributed were presented as median (interquartile range) and compared by nonparametric test. Correlation between different variables was analyzed by Pearson’s correlation analysis and linear regression analysis. Receiver operating characteristic (ROC) curve was used for the independent predictive analysis. Binary logistic regression was used to analyze the relationship between variables and incidence of PCOS. All values of *p* < 0.05 for two-side tests were considered statistically significant.

## Results

### Baseline and Metabolic Characteristics

A total of 216 patients participated in the study, including 104 PCOS patients and 112 women in the control group. The clinical baseline characteristics of all subjects are summarized in **Table 1**. Statistically significant differences in age, estradiol (E2), progestin (P), prolactin (PRL), and thyroid-stimulating hormone (THS) were not found between the two groups (*p* > 0.05). However, compared with the control group, there was a significant increase in body mass index (BMI), luteinizing hormone (LH), testosterone (T), dehydroepiandrosterone (DHEA-s), and anti-Mullerian hormone (AMH), but a decline in follicle-stimulating hormone (FSH) in the PCOS group (*p* < 0.05) as expected, which is due to the clinical features of PCOS. **Table 2** shows the glucose and lipid metabolic characteristics in participants. Low-density lipoprotein (LDL), triglyceride (TG), low-density lipoprotein/high-density lipoprotein (LDL/HDL), apolipoprotein B (ApoB), apolipoprotein B/apolipoprotein AI (ApoB/ApoAI), total cholesterol (TC), and TC/HDL levels were elevated in the PCOS group (*p* < 0.05). On the other hand, fasting blood glucose (FBG), fasting insulin (FINS), and HOMA-IR had no statistically significant difference between the two groups (*P* > 0.05).

**Table 1:** Baseline characteristics and hormones analysis of the population studied.

	Control group (*n* = 112)	PCOS group (*n* = 104)	*p*-value
Age (years)	30.48 ± 4.15	29.75 ± 3.60	0.169
BMI (kg/m^2^)	24.14 ± 3.64	25.35 ± 3.48	0.014^*^
FSH (IU/L)	6.30 ± 1.50	5.60 ± 1.34	0.000^**^
LH (IU/L)	5.08 ± 2.32	8.57 ± 4.75	0.000^**^
E_2_ (pg/ml)	38.37 ± 24.82	38.87 ± 17.26	0.866
P (ng/ml)	0.30 ± 0.28	0.24 ± 0.19	0.090
T (ng/dl)	24.14 ± 11.43	37.37 ± 19.37	0.000^**^
TSH (µIU/ml)	2.26 ± 0.96	2.27 ± 0.97	0.901
PRL (ng/ml)	16.02 ± 5.94	14.42 ± 6.28	0.056
DHEA-s (g/dl)	239.19 ± 85.65	287.57 ± 109.25	0.001^**^
AMH (ng/ml)	4.18 ± 1.94	7.82 ± 3.63	0.000^**^

BMI, body mass index; FSH, follicle-stimulating hormone; LH, luteinizing hormone; E2, estradiol; P, progestin; T, testosterone; TSH, thyroid-stimulating hormone; PRL, prolactin; DHEA-s, dehydroepiandrosterone; AMH, anti-Mullerian hormone.

^*^p < 0.05; ^**^p < 0.01.

**Table 2:** Metabolic analysis of the population studied.

	Control group (*n* = 112)	PCOS group (*n* = 104)	*p*-value
FBG (mmol/L)	5.27 ± 0.37	5.23 ± 0.42	0.464
FINS (µIU/ml)	15.13 (15.23)	16.22 (15.94)	0.209
HOMA-IR	3.46 (4.08)	3.78 (3.94)	0.245
LDL (mmol/L)	2.64 ± 0.67	2.85 ± 0.67	0.030^*^
TG (mmol/L)	1.16 ± 0.67	1.37 ± 0.73	0.026^*^
HDL (mmol/L)	1.36 ± 0.32	1.27 ± 0.35	0.051
LDL/HDL	2.05 ± 0.76	2.37 ± 0.74	0.004^**^
ApoAI (mmol/L)	1.34 ± 0.19	1.32 ± 0.23	0.573
ApoB (mmol/L)	0.82 ± 0.20	0.89 ± 0.19	0.011^*^
ApoB/ApoAI	0.63 ± 0.19	0.70 ± 0.19	0.011^*^
TC (mmol/L)	4.19 ± 0.70	4.40 ± 0.80	0.048^*^
TC/HDL	3.22 ± 0.87	3.64 ± 0.95	0.001^**^

FBG, free blood-glucose; FINS, fasting insulin; LDL, low-density lipoprotein; TG, triglyceride; HDL, high-density lipoprotein; ApoAI, apolipoprotein AI; ApoB, apolipoprotein B; TC, total cholesterol; HOMA-IR = FBG ＊ FINS/22.5.

^*^p < 0.05, ^**^p < 0.01.

### The Expression of ANGPTL4 in Ovarian GCs

**Figure 1:**
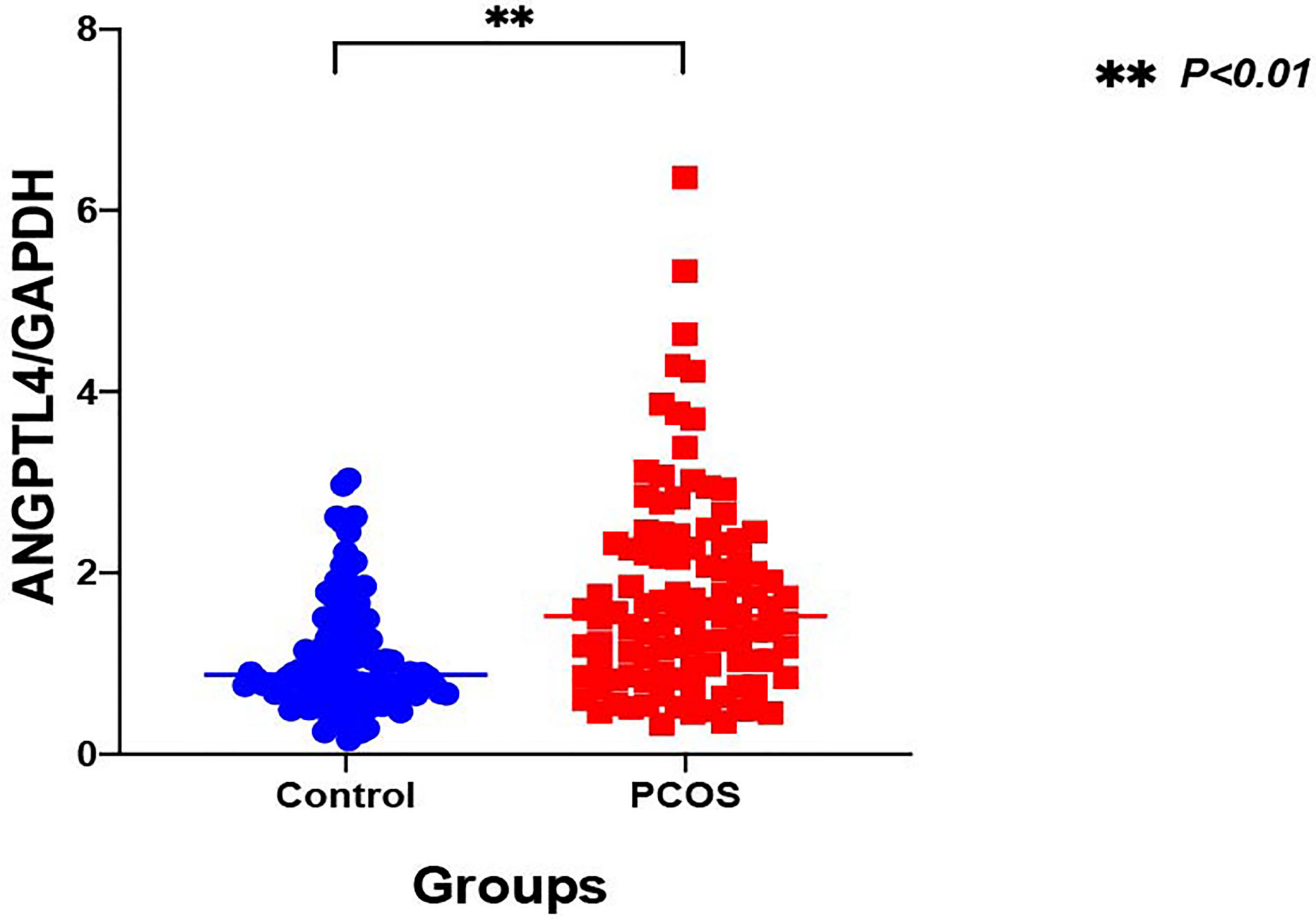
The relative expression of ANGPTL4 in ovarian granulosa cells of PCOS and control patients (**p < 0.001). Data were normalized to GAPDH.

**Figure 2:**
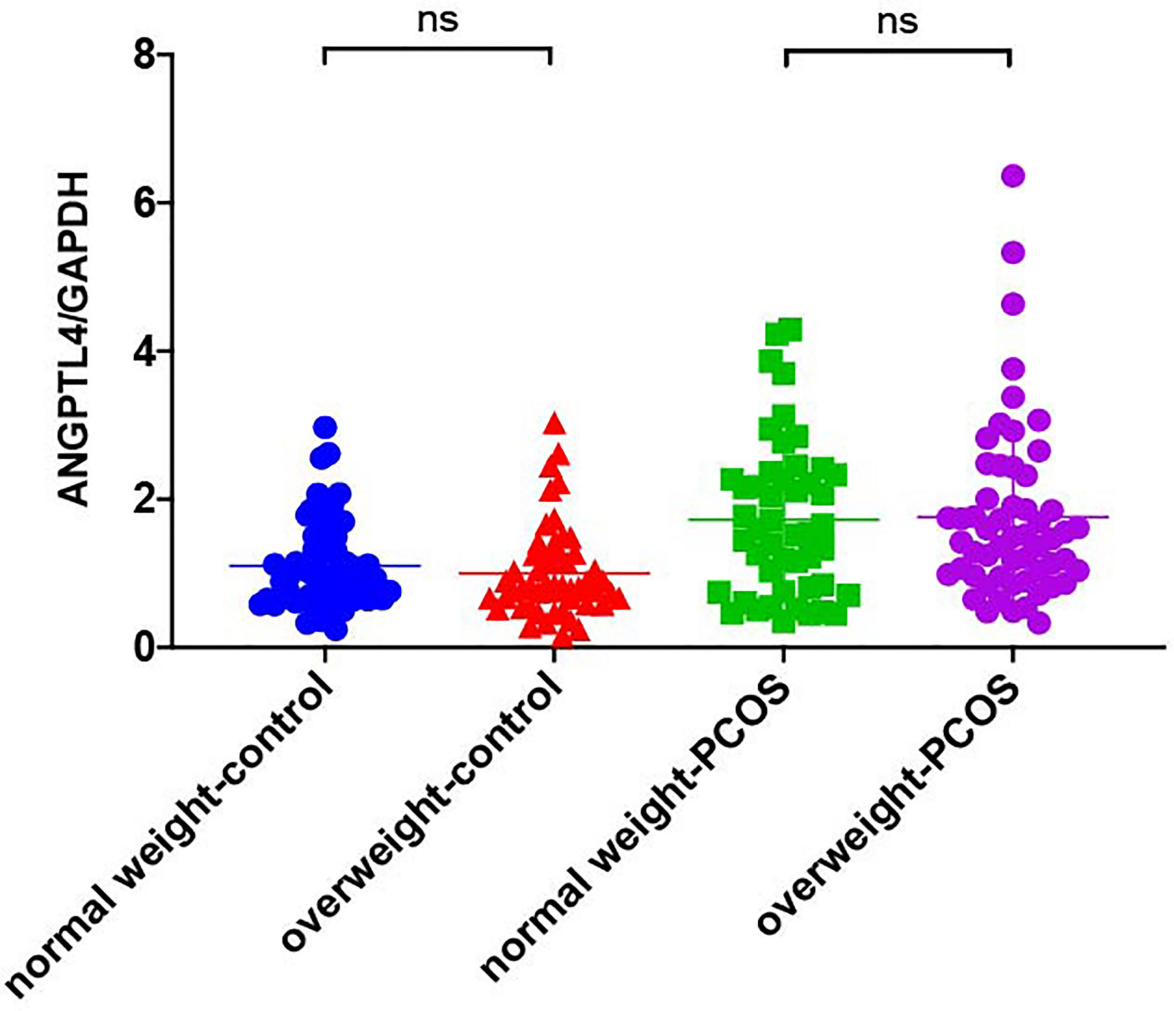
The relative expression of ANGPTL4 in ovarian granulosa cells in subgroups of PCOS and control patients. Data were normalized to GAPDH. "ns" means "no significance".

The expression of ANGPTL4 in ovarian GCs was measured in the two groups by qRT-PCR. Compared with the control group, the expression of ANGPTL4 significantly increased in the PCOS patients [(1.05 ± 0.60) vs. (1.75 ± 1.12), *p* = 0.000] as shown in **Figure 1**. Subgroups as normal-weight group (BMI <25 kg/m^2^) and overweight group (BMI ≥25 kg/m^2^) were set in both control group and PCOS patients. ANGPTL4-expression level had no significant difference between normal-weight group and overweight group regardless of the control group or PCOS group (**Figure 2**).

### The Correlation Between the Expression of ANGPTL4 and the Clinical Characteristics of Patients

**Figure 3:**
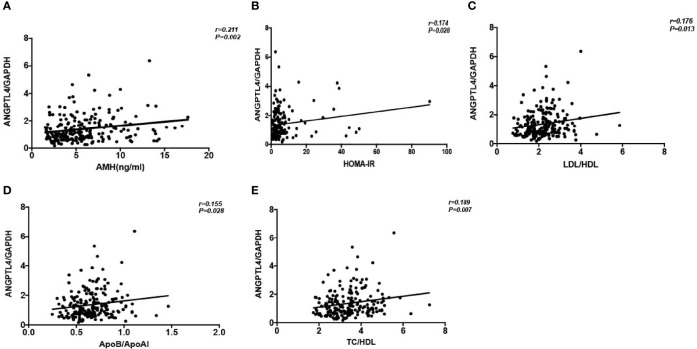
Correlation analysis of ANGPTL4 expression and clinical characteristics. **(A)** AMH (r = 0.211; P = 0.002). **(B)** HOMA-IR (r = 0.174; P = 0.028). **(C)** LDL/HDL (r = 0.176, P = 0.013). **(D)** ApoB/ApoAI (r = 0.155, P = 0.028). **(E)** TC/HDL (r= 0.189, P= 0.007).

We found that the expression level of ANGPTL4 was correlated with AMH (*r* = 0.211, *p* = 0.002), HOMA-IR (*r* = 0.174, *p* = 0.028), LDL/HDL (*r* = 0.176, *p* = 0.013), ApoB/ApoAI (*r* = 0.155, *p* = 0.028), and TC/HDL (*r* = 0.187, *p* = 0.007) positively in all patients in correlation analysis (**Figure 3**). At the same time, the multiple linear regression analysis was conducted to investigate the association between ANGPTL4 expression and clinical characteristics supplementally. Just as shown in **Table 3**, ANGPTL4 expression in ovarian GCs was related to PCOS, FBG, FINS, HOMA-IR, TG, and ApoAI (*p* < 0.05).

**Table 3:** Multiple linear regression analysis of ANGPTL4 relative expression.

	Coefficient			*t*	*p*-value
	Unstandardized		Standardized		
	*β*	SE	*β*		
Constant	7.398	2.631		2.812	0.006^**^
PCOS	0.848	0.205	0.398	3.914	0.000^**^
Age (years)	−0.009	0.022	−0.35	−4.10	0.682
BMI (kg/m^2^)	0.048	0.029	0.166	1.649	0.102
AMH (ng/ml)	0.038	0.028	0.129	1.388	0.168
FBG (mmol/L)	−0.69	0.226	−0.262	−3.051	0.003^**^
FINS (µIU/ml)	−0.027	0.012	−1.072	−2.299	0.023^*^
HOMA-IR	0.140	0.055	1.180	2.532	0.013^*^
LDL (mmol/L)	−1.474	1.581	−0.969	−0.932	0.353
TG (mmol/L)	−0.423	0.183	−0.302	−2.311	0.023^*^
HDL (mmol/L)	0.585	1.517	0.195	0.385	0.701
ApoAI (mmol/L)	−4.466	2.232	−0.876	−2.001	0.048^*^
ApoB (mmol/L)	6.065	3.383	1.222	1.793	0.076
TC (mmol/L)	1.049	1.285	0.785	0.817	0.416

^*^p < 0.05, ^**^p < 0.01.

### Predictive Value of ANGPTL4 Expression in Ovarian GCs for PCOS

**Figure 4:**
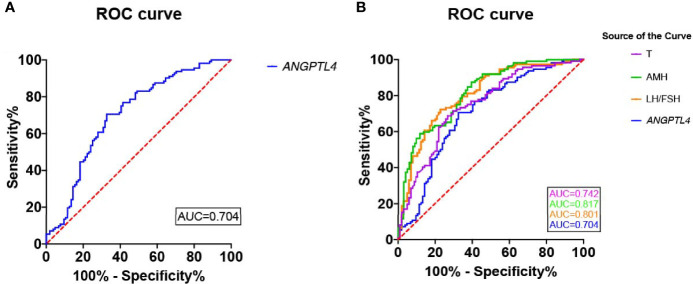
Reciever operating characteristics (ROC) analysis of ANGPTL4 and clinical characteristics for discriminating PCOS. **(A)** Reciever operating characteristics (ROC) analysis of ANGPTL4 for discriminating PCOS. The AUC of ANGPTL4 was 0.704(95% CI 0.633-0.774, P<0.001). **(B)** Reciever operating characteristics (ROC) analysis of 4 clinical characteristics for discriminating PCOS.

**Figure 5:**
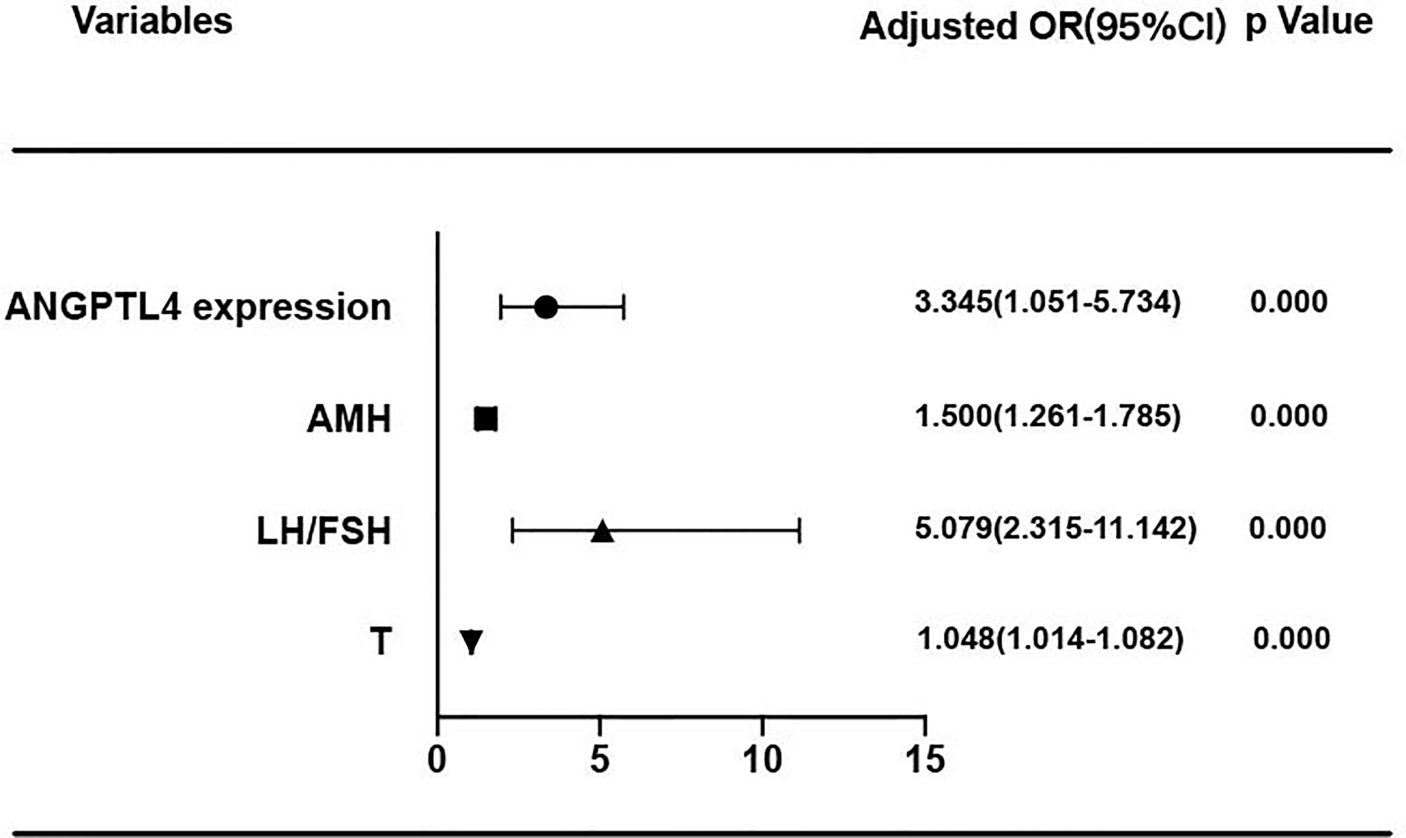
Forest plot of PCOS risk factors. ANGPTL4 expression level were normalized to GAPDH.

In the present study, ROC curve was used to analyze the specificity and sensitivity of ANGPTL4 expression in ovarian GCs for PCOS (**Figure 4**). The area under curve (AUC) of ANGPTL4 expression was 0.704 (95% CI: 0.633–0.774, *p* < 0.001) with sensitivity (67.3%) and specificity (70.5%), compared with T (AUC: 0.742; 95% CI: 0.675–0.808), AMH (AUC: 0.817; 95% CI: 0.761–0.873), and LH/FSH (AUC: 0.801; 95% CI: 0.742–0.860). Binary logistic regression was performed to assess the association between variables mentioned above and incidence of PCOS. The results (**Table 4** and **Figure 5**) showed that the level of ANGPTL4 expression in ovarian GCs could statistically significantly predict the risk of PCOS patients independent of other clinicopathologic variables after adjusting for AMH, LH/FSH, and T (OR: 3.345; 95% CI: 1.951–5.734).

**Table 4:** Binary logistic repression of PCOS risk factors.

Variables	B	SE	Wald	*p*-value	OR	95% CI	
						Lower	Upper
ANGPTL4 expression	1.207	0.275	19.281	0.000^**^	3.345	1.951	5.734
AMH	0.405	0.089	20.924	0.000^**^	1.500	1.261	1.785
LH/FSH	1.625	0.401	16.436	0.000^**^	5.079	2.315	11.142
T	0.046	0.016	7.945	0.005^**^	1.048	1.014	1.082

^**^p < 0.01.

## Discussion

To our knowledge, this is the first research proposing that ANGPTL4 expression level in ovarian GCs had a significant increase in PCOS patients compared with ovulatory women; the AUC of ANGPTL4 expression in GCs supported its important role in incidence of PCOS. In addition, expression level of ANGPTL4 was positively correlated with AMH, HOMA-IR, LDL/HDL, ApoB/ApoAI, and TC/HDL after correlation analysis with the corresponding clinical data in all participants. According to the results of multiple linear regression analysis, ANGPTL4 expression was also related to PCOS, FBG, FINS, HOMA-IR, TG, and ApoAI. In consideration of those results, we just assumed that the ANGPTL4 expression in ovarian GCs might be associated with abnormal glucose and lipid metabolism in PCOS. As we all know, PCOS is a highly heterogeneous endocrine disorder characterized by hyperandrogenism, oligo- or anovulation, and polycystic ovary, and its pathogenesis is debated (19–21). Previous studies have demonstrated that the core etiology and primary endocrine characteristics of PCOS are insulin resistance (IR) and hyperandrogenemia (HA), and their interaction leading to metabolic syndrome, especially dyslipidemia (22–26). Obesity, which was the remarkable feature of metabolic syndrome, increased the risk of insulin resistance (IR) and dyslipidemia, and plays an important role in PCOS (27, 28). In our study, although BMI in PCOS patients were much higher than the control group, the comparison of ANGPTL4 expression among normal-weight and overweight patients showed no difference in both the PCOS group and control group in **Table 3**. AMH are often used as clinical observation indicators reflecting the ovarian reserve (29, 30), and the LH/FSH and T levels were significantly higher in PCOS patients compared with control patients (31, 32). Compared with those characteristics related to PCOS, the expression level of ANGPTL4 in ovarian GCs might be an independent factor which affected incidence of PCOS according to results of ROC curve and binary logistic regression analysis.

As a member of the angiopoietin-like protein family, ANGPTL4 has been extensively investigated, and its involvement in physiological and pathological conditions including energy metabolism, tumorigenesis, vascular homeostasis, and inflammation has been reported (33–37). One of the extensively investigated roles of ANGPTL4 was its effect in lipid metabolism, specifically in regulating LPL activity to clear TGfrom the circulation (10). In addition, a recent study demonstrated that ANGPTL4 knockout mice markedly improved glucose tolerance with increased insulin levels (38). Güneş et al. (13) revealed that there was a significant increase in the level of serum ANGPTL4 compared with the control group and IR was significantly associated with ANGPTL4 concentrations in patients. Those were corresponding to the results in our study that the ANGPTL4 expression in ovarian GCs was higher in PCOS, and glycolipid characteristics such as FBG, FINS, HOMA-IR, TG, and ApoAI were also associated with ANGPTL4 expression. In view of the result of our study, ANGPTL4 might participate in the glucose and lipid metabolism in the ovarian surroundings, which might affect the occurrence and development of PCOS. In addition, ANGPTL4 also acts as an apoptosis survival factor of vascular endothelial cells. It plays a key role in the late stages of folliculogenesis and participates in providing oxygen and nutrients for growing follicles (39, 40). Our study showed a positive correlation between ANGPTL4 expression and AMH and indicated its possible impact on follicular development. All of our findings were preliminary, and more research of mechanisms is expected later.

High expression of ANGPTL4 in PCOS and its possible association with multiple glucose and lipid metabolism characteristics suggests that ANGPTL4 expression level might play an important role in pathogenesis and development of PCOS. Our findings also raise a series of important new questions. What is the mechanism underlying ANGPTL4 expression in PCOS? What is the role of ANGPTL4 signaling for metabolism in the ovarian GCs? All these questions have not yet been reported.

The main strength of the study is that we focused on the relationship between ANGPTL4 and PCOS and linking them together has not been done previously, and the ANGPTL4 expression in ovarian GCs might be a risk factor for the occurrence and development of PCOS by participating in glucose and lipid metabolism. This has not been confirmed before. Our study has several limitations as below. Weakly positive correlation between ANGPTL4 expression level and glycolipid metabolism just indicates possible relation without enough evidence. Small sample size and retrospective study design requires large sample and further basic experimental research to investigate the pathophysiologic progress of PCOS.

## Conclusion

Our study showed that differential expression of ANGPTL4 in ovarian GCs was identified between PCOS patients and control women, and its association with glucose and lipid metabolism showed that the high expression of ANGPTL4 might be an independent factor of the incidence of PCOS and played a role in metabolism and pathogenesis of PCOS.

## Data Availability Statement

The raw data supporting the conclusions of this article will be made available by the authors, without undue reservation.

## Ethics Statement

The studies involving human participants were reviewed and approved by the Ethics Committee of the Reproductive Medicine of Shandong University. The patients/participants provided their written informed consent to participate in this study. Written informed consent was obtained from the individual(s) for the publication of any potentially identifiable images or data included in this article.

## Author Contributions

YS conceived and designed this study. QJ contributed to experiments, statistical analysis, interpretation of data, and drafting of the manuscript. YP, YZ and PL performed statistical analysis and participated in the discussion. WW, TS, and GW acquired the data. YB analyzed and interpreted the data. YS participated in the discussion and critically revised the manuscript. All authors read and approved the final manuscript.

## Funding

This study was supported by National Key R&D Program of China (2021YFC2700404, 2018YFC1003202) and Taishan scholar project special funds (No. ts201712103).

## Conflict of Interest

The authors declare that the research was conducted in the absence of any commercial or financial relationships that could be construed as a potential conflict of interest.

## Publisher’s Note

All claims expressed in this article are solely those of the authors and do not necessarily represent those of their affiliated organizations, or those of the publisher, the editors and the reviewers. Any product that may be evaluated in this article, or claim that may be made by its manufacturer, is not guaranteed or endorsed by the publisher.
